# Artificial Intelligence–based Quantification of Pleural Plaque Volume and Association With Lung Function in Asbestos-exposed Patients

**DOI:** 10.1097/RTI.0000000000000759

**Published:** 2023-11-01

**Authors:** Kevin B.W. Groot Lipman, Thierry N. Boellaard, Cornedine J. de Gooijer, Nino Bogveradze, Eun Kyoung Hong, Federica Landolfi, Francesca Castagnoli, Nargiza Vakhidova, Illaa Smesseim, Ferdi van der Heijden, Regina G.H. Beets-Tan, Rianne Wittenberg, Zuhir Bodalal, Jacobus A. Burgers, Stefano Trebeschi

**Affiliations:** * Department of Radiology; † Department of Thoracic Oncology, Netherlands Cancer Institute, Amsterdam; ‡ Technical Medicine, University of Twente, Enschede; § GROW School for Oncology and Developmental Biology, Maastricht University Medical Center, Maastricht; §§ Department of Robotics and Mechatronics, University of Twente, Enschede, The Netherlands; ∥ Academic Pridon Todua Medical Center, Research Institute of Clinical Medicine, Tbilisi, GA; ¶ Seoul National University Hospital, Seoul, South Korea; # Radiology Unit, Sant’Andrea Hospital, Sapienza University of Rome, Rome, Italy; ** Department of Radiology, University of Brescia, Brescia, IT; †† Department of Radiology, Royal Marsden Hospital, London, UK; ‡‡ Division of Radiotherapy and Imaging, The Institute of Cancer Research, London, UK; ∥∥ Faculty of Health Sciences, University of Southern Denmark, Odense, DK

**Keywords:** pleural diseases tomography, x-ray computed respiratory function tests asbestos artificial intelligence

## Abstract

**Purpose:**

Pleural plaques (PPs) are morphologic manifestations of long-term asbestos exposure. The relationship between PP and lung function is not well understood, whereas the time-consuming nature of PP delineation to obtain volume impedes research. To automate the laborious task of delineation, we aimed to develop automatic artificial intelligence (AI)–driven segmentation of PP. Moreover, we aimed to explore the relationship between pleural plaque volume (PPV) and pulmonary function tests.

**Materials and Methods:**

Radiologists manually delineated PPs retrospectively in computed tomography (CT) images of patients with occupational exposure to asbestos (May 2014 to November 2019). We trained an AI model with a no-new-UNet architecture. The Dice Similarity Coefficient quantified the overlap between AI and radiologists. The Spearman correlation coefficient (*r*) was used for the correlation between PPV and pulmonary function test metrics. When recorded, these were vital capacity (VC), forced vital capacity (FVC), and diffusing capacity for carbon monoxide (DLCO).

**Results:**

We trained the AI system on 422 CT scans in 5 folds, each time with a different fold (n = 84 to 85) as a test set. On these independent test sets combined, the correlation between the predicted volumes and the ground truth was *r* = 0.90, and the median overlap was 0.71 Dice Similarity Coefficient. We found weak to moderate correlations with PPV for VC (n = 80, *r* = −0.40) and FVC (n = 82, *r* = −0.38), but no correlation for DLCO (n = 84, *r* = −0.09). When the cohort was split on the median PPV, we observed statistically significantly lower VC (*P* = 0.001) and FVC (*P* = 0.04) values for the higher PPV patients, but not for DLCO (*P* = 0.19).

**Conclusion:**

We successfully developed an AI algorithm to automatically segment PP in CT images to enable fast volume extraction. Moreover, we have observed that PPV is associated with loss in VC and FVC.

Pleural plaques (PPs), a specific manifestation of asbestos exposure, often appear on the parietal pleura as localized hyalinized collagen fibers in calcified or noncalcified forms.^1–3^ The exact mechanism of PP formation remains unclear.^2,4^ However, the likelihood of developing PP is associated with the duration and cumulative exposure to asbestos.^5^ Despite this, PP can also form after minimal exposure.^6^

Patients with PP are typically asymptomatic.^7^ Discrepancies exist between a systematic review indicating no statistically significant association between PP and pulmonary function test (PFT)^8^ and a study demonstrating a small, statistically significant impact on lung function.^9^ Thoracic computed tomography (CT) enables PP extension measurement with excellent intraobserver reproducibility (intraclass correlation: 0.98) and good interobserver variability (intraclass correlation: 0.93).^10^ However, manual segmentation of volume is time-consuming and impractical for large population studies or clinical workflow integration.^11^ Consequently, the impact of PP on lung function remains inconclusive.

Public health policies in many countries provide financial support for patients with mesothelioma or asbestosis after occupational asbestos exposure.^12,13^ However, few policies consider PPs due to the lack of evidence supporting a clinically significant loss in lung function.^8^ Even with confirmation, manual volumetric assessment would be incompatible with the current radiologic workflow.^11^

An alternative method for segmentation and volume quantification is needed to facilitate extensive population studies and clinical implementation of pleural plaque volume (PPV) measurements. This method should be fast, accurate, and reproducible. Artificial Intelligence (AI)–based automated segmentation could provide a potential solution by learning to identify patterns in CT scans and yield a volumetric measurement of PP in seconds.

This study aims to develop an AI algorithm for the automatic segmentation of PP and examine the relationship between PP and lung function impairment. The resulting algorithm will enable researchers to investigate the correlation between PPV and lung function, providing a proof of concept for a clinically compatible, quantitative PPV test.

## MATERIALS AND METHODS

### Data Sets

We performed a retrospective analysis on a data set of people applying for state financial support, between May 2014 and November 2019.^12^ This data set is comprised of a cohort of applicants who are required to submit a CT scan acquired from their respective local hospital, along with a PFT. The data set was collected by the K.B.W.G.L and C.J.d.G. Inclusion criteria were fully imaged lungs on CT with slice thickness ≤5 mm. Thoracic CTs were collected from multiple hospitals across the country, resulting in heterogeneous data (median, CI): voltage (120 kVp, 118.7 to 121.3), tube current (194 mA, 177.4 to 210.6), and slice thickness (3 mm, 2.85 to 3.14). Vendors, reconstruction kernels, and contrast usage are listed in Table 1. CT scans were acquired with breath-hold at mid-respiratory or inspiratory volume. As part of the financial support compensation procedure, 3 independent pulmonologists determined whether significant fibrosis was present, defined as >5% of lung parenchyma.^12^

**TABLE 1:** Technical Parameters of the Included CT Scans, Filtered by Manufacturer and Reconstruction Kernel

Manufacturer	Convolution kernel	Count
GE Medical Systems	BONE	1
GE Medical Systems	BONEPLUS	10
GE Medical Systems	CHST	7
GE Medical Systems	LUNG	27
GE Medical Systems	SOFT	1
GE Medical Systems	STANDARD	7
Philips	A	4
Philips	B	41
Philips	C	15
Philips	E	3
Philips	IMR1,SharpPlus	3
Philips	L	34
Philips	YA	3
Philips	YC	10
Philips Medical Systems	5	1
SIEMENS	B30-B45, I30-I45	99
SIEMENS	B60-B80, I50-I70	51
SIEMENS	Bl57	3
SIEMENS	Bl64	1
SIEMENS	Br40	2
SIEMENS	Br69	1
SIEMENS	Ub44u	1
TOSHIBA	BODY	1
TOSHIBA	FC02-FC35	42
TOSHIBA	FC51-FC86	30
TOSHIBA	LUNG	1
Unknown	—	23

Applicants signed a written informed consent that their data could be used for systematic analyses. The project was approved by the Institutional Scientific Board (IRBd19-136) and performed in accordance with the Declaration of Helsinki. The de-identification process for the data was executed in compliance with the DICOM standard, utilizing proprietary software developed within the institution.

PFT for patients in the study data set were retrospectively retrieved. When recorded, the parameters were: vital capacity (VC), forced vital capacity (FVC), and diffusing capacity for carbon monoxide (DLCO). The PFT data were acquired from spirometry tests in an upright position with expiratory measurements and converted to percentage predicted values following Global Lung Function Initiative 2012 reference equations for spirometry.^14,15^

### Segmentation Procedure

A team of 5 board-certified radiologists (T.N.B., N.B., E.K.H., F.L., and F.C.) manually segmented the PPs using 3D Slicer v4.11,^16^ with the workload was split equally among them. The time per segmentation was not recorded. However, readers mentioned that segmentation took 30 to 60 minutes per scan. Calcified and noncalcified portions of the plaques were both segmented as one single segment. One technical physician^17^ with 2 years of experience in thoracic CT imaging (K.B.W.G.L.) reviewed all CT scans and segmentations and forwarded inconsistent segmentations to another team of radiologists (T.N.B., N.V., I.S., R.W.). They adjusted the segmentations of suboptimal quality (segmentation artifacts, missing plaques, etc). To analyze the AI segmentation performance of calcified versus noncalcified plaques, we set an empirical threshold of 120 HU to differentiate between them as a postprocessing step.

### Design of the AI Algorithm

**FIGURE 1:**
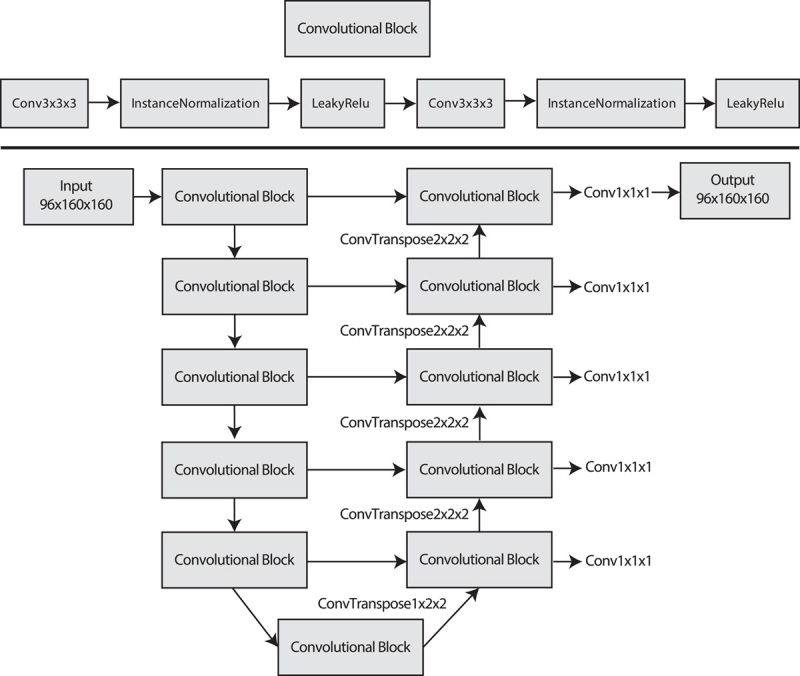
Schematic overview of the nnUNet architecture based on the characteristics of the PP data set. Top: details of the convolutional block used throughout the model. Bottom: overview of the total architecture. The input size (slices, *y, x*) is equal to the output size, referred to as the patch size.

We implemented the AI algorithm following the design of the no-new-UNet (nnUNet).^18^ This represents the state-of-the-art in image segmentation, with the algorithm leveraging several preprocessing techniques and training procedures. The nnUNet system automatically determines the optimal Convolutional Neural Network architecture and other hyperparameters based on the characteristics of the data set (ie, the thoracic CT scans). During training, a “patch” equal to the input size of the model is retrieved from the CT scan, and the algorithm iterates over these patches until the entire CT scan is analyzed. The configuration chosen for the architecture was 3D full resolution with training procedure (trainer) nnUNetTrainerV2. A schematic overview of the architecture is shown in Figure 1.

### Data Preprocessing and Training Procedure

We split the data set into training (n = 337, 80%) and a test set (n = 85, 20%), based on a random reproducible split. All CT scans were resampled to (0.71, 0.71, 1; x, y, z) spacing with a patch size of (160, 160, 96; x, y, z). The training procedure consisted of 1000 epochs with a batch size of 4. The loss function was a combination of the dice loss and cross-entropy. To test whether the ensemble 5-fold cross-validation outperformed the single model trained on all data, we ran experiments with and without internal cross-validation.

### AI Model Evaluation

The segmentation performance of the trained AI model was evaluated using the Dice Coefficient Score (Dice Similarity Coefficient [DSC]), which is a quantitative measure to determine the overlap between the predicted segmentation by the AI, and the ground truth. The higher the overlap between the two, the better the performance of the AI. In addition to the DSC, we calculated the correlation between the volume predicted by the AI model and the ground truth volume derived from the segmentation of the expert readers. This allowed us to identify possible systematic errors in the model and the presence of outliers. To test whether the AI can measure the PPV in the CT scans correctly, we used the different percentiles to convert the segmentation task to a classification problem. Here, we monitor whether the AI classified the CT scans as containing a higher or lower PPV than the cut-off, compared with the radiologist’s segmentation.

### Lung Volume Assessment

The lung volume was quantified using an external AI model for lung segmentation.^19^ The output generated by the model was then manually reviewed and corrected, if needed, by K.B.W.G.L. using the 3D Slicer software. This particular AI model was selected because it demonstrated adequate accuracy and robustness during internal evaluation, generalizing reasonably well to fibrotic tissue. This attribute made corrections for fibrotic lungs more feasible compared with other models, establishing it as a reliable choice for our research study.

### Association Between PP and Decreased Lung Function

Due to the slow growth rate of PPs,^2^ we do not expect significant volume differences over distinct periods of months. As a result, we performed the analysis using PFT data from patients within 1 year, measured from the date of the CT scan. To determine whether an increase in PPV is associated with decreased lung function, we calculated the correlation between PPV and lung function parameters and tested for significant differences in lung function for groups at different cut-offs of PPV, namely the 25th, 50th, and 75th percentiles. Given that the lung function parameters are normalized in percent predicted values, we normalized the PPV as well through the total lung volume of the patient. The normalized PPV consisted of the PPV divided by the lung volume of the patient. Differences between FVC and VC may indicate air trapping or small airway collapse. Therefore, we tested this difference versus the PPV. Patients with diffuse fibrosis were excluded from a correlation between lung function and the volume of PPs as fibrosis is a confounding variable.^20^

### Statistical Analysis

As the PPV and PFT results were not normally distributed, the Spearman *r* was calculated. We applied the Mann-Whitney *U* test to test the differences between the cross-validated model and the single model on the same test set. Differences in PFT between groups with different PPVs were assessed through the Wilcoxon signed-rank test. The 95% CIs were calculated through bootstrapping with replacement. Bonferroni correction was applied when multiple tests were conducted. Bonferroni corrections were applied to account for the 3 distinct tests conducted across various quartiles of PPV in relation to PFT. This adjustment resulted in a significance level (α) of P<0.05/3=0.017 .

## RESULTS

### Study Cohort

We retrospectively collected (n = 523) applications for asbestosis government support. The median age of the applicants was 75 years (interquartile range [IQR]: 69 to 80), and applicants were almost exclusively male (2 females, 0.4%). Applications were excluded due to the absence of CT scans (n = 74) and any PP (n = 27), yielding a total data set of 422 CT scans (n = 303 with contrast). All scans were segmented by radiologists and reviewed. Three PFTs were collected when available: VC (n = 393, median 79, IQR: 64 to 96), FVC (n = 398, median 78, IQR: 64 to 95), DLCO (n = 408, median 57, IQR: 44 to 71). There was no statistically significant difference observed between the total cohort and the cohort after exclusion in terms of age (median: 73 vs 74 y, *P* = 0.39) or PFTs (VC: 74% vs 74%, *P* = 0.47; FVC: 79% vs 79%, *P* = 0.39; DLCO: 55% vs 55%, *P* = 0.43).

### Interrater Variability of Adjusted Cases

**FIGURE 2:**
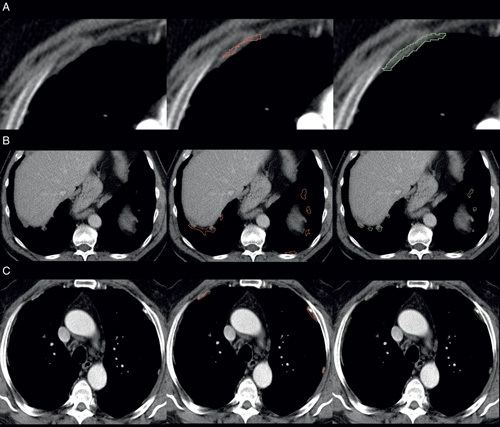
Several examples of adjusted segmentation after inconsistencies were noted. The first column is the CT scans, the second column is the segmentation of the first radiologist, and the third column is the revised segmentation. A, CT scans in the axial plane with contrast; PP only partially segmented. B, CT scans in axial plane without contrast; PPs segmented on lung window, leading to overestimation of the volume. C, CT scans in the axial plane with contrast; missed PP.

In total, 68 segmentations were admitted for review due to inconsistencies, and all of them were adjusted after inspection by the radiologist. For these adjustments, we observed the following medians: DSC of 0.61, a sensitivity of 0.48, an initial volume of 37.4 cm^3^, and a corrected volume of 81.8 cm^3^. Figure 2 shows several examples of adjusted annotations, with reasons such as partly segmented PPs, using lung window during segmentation, and missing PPs.

### Cross-validation Versus Single Model

The first experiment consisted of 5-fold cross-validation (standard nnUNet procedure, ensemble model) and another experiment of a single training procedure with all training data (single model). The ensemble model reached a median DSC of 0.70 (0.66 to 0.73), on par with the single model with a median DSC of 0.70 (0.69 to 0.74), *P* = 0.60, evaluated on the same independent test set.

### Single Models on Different Test Sets

We trained multiple models to study the influence of the chosen test set, with each patient in the independent test set once. Therefore, the nnUNet architecture was trained five times, each with a different, random, reproducible split. We made all trained algorithms available at https://github.com/nki-radiology/pleural-plaques.

**FIGURE 3:**
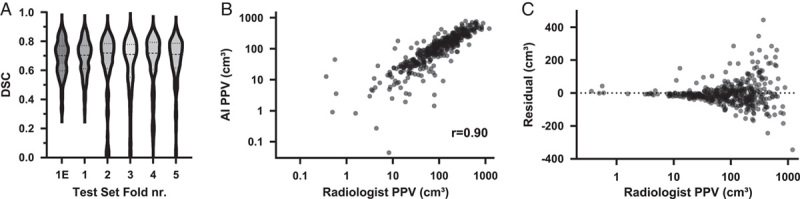
A, DSC distribution over the AI models, each with a different 20% as a test set, including the ensemble method (1E). B, Correlation between the combined test sets of AI-predicted PPV and the radiologist-segmented PPV. C, The *x*-axis denotes the radiologist-segmented PPV, and the *y*-axis represents the difference between the radiologist and the AI. The higher the radiologists’ segmented volume, the larger the difference.

**FIGURE 4:**
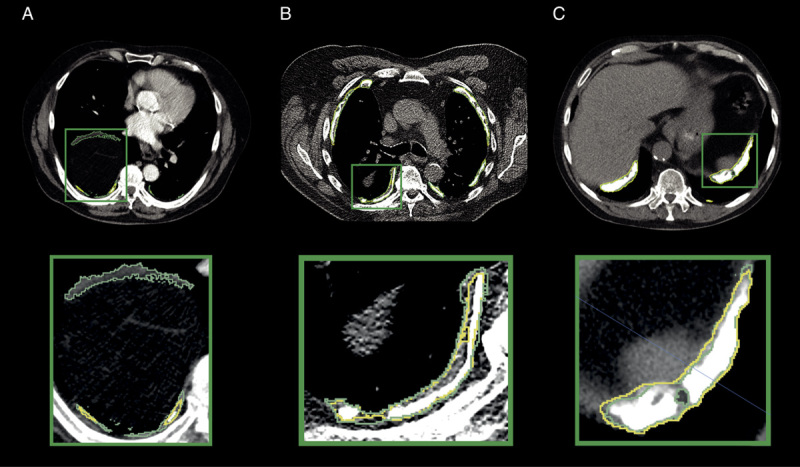
A, CT scans in the axial plane with contrast; an example of the lower value of DSC between AI in yellow outline, and radiologist in green outline (DSC = 0.43). B, CT scans in axial plane without contrast; average segmentation performance (DSC = 0.75). C, CT scans in axial plane without contrast; well-segmented plaques on the diaphragm (DSC = 0.85).

All (n = 5) AI models yielded similar performances over the individual scans in the test set with a median DSC of 0.70 (0.69 to 0.74), 0.72 (0.67 to 0.73), 0.71 (0.67 to 0.73), 0.72 (0.69 to 0.75), and 0.71 (0.68 to 0.75) (Fig. 3A, Table 2). No statistically significant differences existed between the test set results (*P* > 0.05). Combining the predictions on all test sets, the median DSC is 0.71 (0.70 to 0.73). The overall median sensitivity on the combined test set is 0.78 (0.74 to 0.80). In terms of PPV, the difference between the volume segmented by experts (median: 104.0 cm^3^, CI: 86.9 to 119.9 cm^3^) versus the AI models (median: 121.8 cm^3^, CI: 101.6 to 136.1 cm^3^) did not reach the level of statistical significance (*P* = 0.09). The mean absolute error was 29.7 cm^3^ (CI: 23.5 to 35.7). AI-predicted volume and the segmented volume showed a strong correlation (spearman *r =* 0.90, CI: 0.88 to 0.92, *P* < 0.001; Fig. 3B). The difference between radiologists and AI segmentation increased as the segmented volume of the radiologists increased (Fig. 3C). We visualize several cases with different quality of segmentation in Figure 4A–C. Segmentation of the calcified PPs yielded a DSC of 0.92 (0.91 to 0.93) and a sensitivity of 0.96 (0.95 to 0.97), with a significant difference (*P* < 0.0001) between AI-predicted volume of 27.38 cm^3^ (21.13 to 32.40 cm^3^) and the expert derived volume of 23.72 cm^3^ (19.62 to 29.75 cm^3^). The noncalcified part of PP yielded a DSC of 0.62 (0.60 to 0.64) and a sensitivity of 0.69 (0.66 to 0.72), where the difference between AI predicted volume of 81.58 cm^3^ (70.54 to 93.81 cm^3^) and the expert volume of 74.34 cm^3^ (61.15 to 89.31 cm^3^) was not statistically significant (*P* = 0.28). The AI was able to classify all percentiles with excellent performance (25th percentile: area under the curve [AUC] = 0.94 [0.92 to 0.97]), *P* < 0.0001, 50th percentile: AUC = 0.95 (0.93 to 0.97), *P* < 0.0001, 75th percentile: AUC = 0.95 (0.93 to 0.97) *P* < 0.0001).

**TABLE 2:** Metrics of Each of the Individual-trained Models Reported in the Median and 95% CI

Model	DSC	Sensitivity	Label volume	Pred volume (cm³)	Absolute error volume
1	0.70 (0.69-0.74)	0.78 (0.71-0.83)	102.1 (75.5-185.5)	104.7 (80.5-136.5); *P* < 0.0001	23.0 (17.4-30.4)
2	0.71 (0.67-0.73)	0.75 (0.71-0.81)	102.4 (75.3-125.9)	98.4 (80.7-153.1); *P* < 0.0001	41.4 (22.6-53.6)
3	0.72 (0.69-0.75)	0.78 (0.70-0.84)	87.58 (63.9-112.6)	104.9 (70.1-144.5); *P* < 0.0001	29.7 (21.9-37.7)
4	0.72 (0.67-0.73)	0.77 (0.69-0.81)	87.43 (67.3-140.0)	119.6 (72.3-152.5); *P* < 0.0001	28.1 (17.5-46.1)
5	0.71 (0.68-0.75)	0.77 (0.74-0.83)	143.7 (107.7-179.9)	161.8 (121.8-186.7); *P* < 0.0001	32.0 (22.0-37.9)

Label volume indicates PP volume segmented by the radiologists; Pred volume indicates volume segmented by the AI model.

*P* value is calculated with a Wilcoxon paired test between label volume and pred volume.

### Comparison With PFTs

**FIGURE 5:**
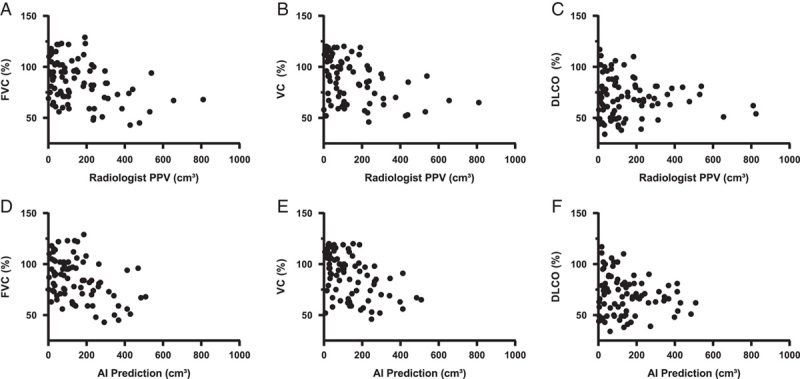
A, Scatterplot of the FVC versus the PPV by the radiologist. B, VC versus PPV. C, Diffusing DLCO versus PPV. D, Scatterplot of the FVC versus the PPV by the AI models. E, VC versus PPV by AI. F, DLCO versus PPV by AI.

The data set contained 188/423 patients without diffuse fibrosis, of which 106 patients had a PFT within a year of the CT date. We collected the VC (n = 80), FVC (n = 82), and DLCO (n = 84), where 50 patients had complete data for all 3 measurements. Figure 5A–C shows the relation of each parameter to the PPV segmented by the radiologists, whereas Figure 5D–F shows the relation with AI segmented volume. PPV segmented by the radiologists was moderately negatively correlated with VC (*r* = −0.40, CI: −0.54 to −0.22, *P* = 0.0003) and FVC (*r* = −0.38, CI: −0.52 to −0.21, *P* = 0.0005), but not correlated with DLCO (*r* = −0.09, CI: −0.25 to 0.08, *P* = 0.39). All panels show a nonlinear relation between the PPV and lung function, where a high PPV suggests an association with low lung function values. The normalized PPVs by lung volume were moderately negatively correlated for VC (*r* = −0.45, CI: −0.60 to −0.28, *P* < 0.0001) and FVC (*r* = −0.42, CI: −0.57 to −0.25, *P* < 0.0001), but no statistically significantly correlation was observed for DLCO (*r* = −0.11, CI: −0.26 to 0.05, *P* = 0.30). No correlation was found between normalized PPV and the difference between VC and FVC (*r* = 0.00, CI: −0.20 to 0.21, *P* = 0.60). By splitting the PPV distribution into the different quartiles (45, 106, and 229 cm^3^), we observed a statistically significant lower VC and FVC for the higher PPV group (Table 3). DLCO did not yield any statistically significant difference.

**TABLE 3:** Difference in Lung Function Test Based on Several Cutoffs of PPV

Lung function	Percentile	PPV	n upper	n lower	Mean upper	Mean lower	*P*
VC	25	34.7	60	20	84.6	96.3	**0.016**
VC	50	98.8	40	40	80.3	94.6	**0.001**
VC	75	228.2	20	60	73.6	92.1	**<0.001**
DLCO	25	37.3	63	21	69.5	71.0	0.49
DLCO	50	103.5	42	42	67.4	72.4	0.19
DLCO	75	225.5	21	63	66.8	70.9	0.27
FVC	25	41.3	61	21	83.2	91.4	0.055
FVC	50	104.8	41	41	81.0	89.6	0.037
FVC	75	233.3	21	61	69.0	90.6	**<0.001**

Bonferonni correction has been applied (significance level = 0.017).

Bold values indicate a statistically significantly result after correction.

## DISCUSSION

In this study, we proposed an AI algorithm for fast, automatic assessment of PPV. Our goal was to design an automated segmentation model for PPs to enable further research on the impact of PPV on patients. The segmentation results suggested an adequate ability of the algorithm to replicate the expert reader’s segmentation and estimate the PP total volume. We showcased how the algorithm can enable researchers to test the relation between PPV and PFTs in a data set of former asbestos workers applying for government support. A nonlinear association between VC, FVC, and both PPV and PPV corrected for lung volume was observed, exceeding the relation found in current literature.^8,9,11,21^

To the best of our knowledge, we are the first study providing an automatic segmentation tool for future research in PP and asbestos exposure. In our study, we use state-of-the-art 3D segmentation and a larger data set to obtain higher accuracy and precision and share it freely online for the scientific and medical community to use. We showed that the ensemble method did not outperform a single model training procedure for this data set. Interestingly, the other folds yielded a nonsignificant higher median DSC than the first fold (both ensemble and single model) but also produced outliers with DSC scores between 0 and 0.2. No outliers would have been reported if only the first-fold results were published. However, by running multiple experiments on different test sets, we showed that four out of 5 folds yielded outliers, leading to a moderate median DSC over all test sets. A potential reason for the difference between AI and expert segmentation is the different acquisition and reconstruction protocols in the data set (Table 1), where the AI model does not generalize sufficiently. Having multiple radiologists independently delineate PP without consensus could be another reason why each radiologist would segment PP differently. Although the AI outperformed the interobserver variability of the worst annotated cases (the revisions), the study could not analyze the overall interobserver variability, nor the intraobserver variability.

In related work, another study investigated PP segmentation on 5 mm slices with deep learning^22^ but did not investigate its association with PFT. A study that investigated the association included 26 patients, who were divided into 3 groups of <10 mL, 10 to 20 mL, and >20 mL PPV, where no statistically significant differences were found between the groups in terms of lung function values.^11^ We defined different cutoffs to determine the high and low PPV groups. Our lowest cutoff of the 25th percentile was 39.6 cm^3^ (or mL) for VC, where a similar study defined the highest volume group of plaques as >20 cm^3^,^11^ representing a substantial shift in our understanding of the extent of the disease. Another study measured the PPV of 75 patients on 3 axes and could not correlate this volume to lung function, exercise capacity, and cumulative asbestos exposure.^21^ The full volumetric measure, instead of a surrogate measure of the 3 longest diameters,^21^ seems to be a key point in the understanding of the relationship between PPs and lung function.

To demonstrate the usability of the algorithm to study lung function, we presented an example with FVC, VC, and DLCO. In our showcase, significant differences were observed in PPV in relation to both FVC and VC. The difference between FVC and VC is the forcefulness of exhalation. When discrepancies occur, it could indicate airway resistance, for example. The results suggest that PPV does not lead to differences between FVC and VC, as we observed no correlation. The total lung capacity is the volume of gas in the lung at the end of full inspiration. A decreased total lung capacity reflects a restrictive lung disorder. It is the sum of the inspiratory reserve volume (IRV), tidal volume, expiratory reserve volume, and residual volume. The (F)VC is the volume of exhaled air after a maximal inspiration, consisting of the tidal volume, expiratory reserve volume, and IRV. A reduction in (F)VC can indicate restrictive lung disease, which can be categorized as an intrapulmonary (parenchymal) disease, such as lung fibrosis. Therefore, a possible cause of the observed decreased (F)VC in our patient group is that PPV reduced the expansion of the lungs, which decreases the IRV. DLCO (gas exchange) is less affected by total air inhalation,^23^ which may explain the non-significant relation.

If, by means of our algorithm, further studies are able to unveil the relation between quality of life and extent of PP, and to confirm a decrease in quality of life, financial compensation programs for patients with PPs in more countries might arise. For example, the United Kingdom canceled its compensation for PPs in 2007 due to the absence of evidence that PPs impeded lung function.^24^ Moreover, if, in the future, governments decide that a certain PPV lung function loss is endorsed for compensation, our model might be used to detect whether that PPV threshold is reached. This avoids the labor-intensive and time-consuming work of the radiologists who would otherwise have to segment the plaques. In such a workflow, a radiologist should evaluate the segmentation of the AI model and approve or adjust it for finalization. An intuitive graphical user interface to interact with the AI segmentation should, therefore, be developed.

From a clinical perspective, a completely automated and precise model has the potential to monitor alterations in PPV over time. Notably, if specific areas of PP demonstrate accelerated growth, it may be suggestive of pleural mesothelioma.^25^ Given the typical late-stage detection of mesothelioma,^26^ this method might provide an active surveillance approach for patients with PP who have been exposed to asbestos.

There are limitations to this study. First, a substantial portion of the PP segmentations was revised, which indicates a high interobserver variability among the radiologists, unlike the findings of another study that found minimal interobserver variability.^10^ Radiologists reported that in CT scans with suboptimal quality, it was hard to distinguish PPs from other structures, leading to interobserver variance. CT scans at mid-respiratory and inspiratory volume were included, which may bias the lung volume measurement, and, therefore, subsequently the PPV versus lung volume ratio. The suboptimal DSC of the otherwise excellent performing nnUNet architecture could have several reasons: poor generalizability over different reconstruction kernels, vendors, or resolutions; high interrater variability among readers, leading to inconsistent segmentations as ground truth; or suboptimal segmentation guidelines (eg, window selection, decision-making in uncertain cases regarding whether to segment or not). Furthermore, in our showcase, we could not correct for confounders in the correlation between the lung function parameters and the volume. Moreover, lung function parameters were already in percentage of predicted value, resulting in a complex comparison with an absolute measure, such as PPV. Therefore, an additional analysis was performed where the PPV was corrected for lung volume. While we did exclude patients with substantial pulmonary fibrosis, lung function parameters for other confounders (eg, asbestos exposure, Body Mass Index, and smoking^8^) could not be corrected as that information was unknown. An extensive analysis of the correlation between lung function and PP extension is beyond the scope of this study. The algorithm is available online, for other researchers to use, replicate our results, and study the influence of confounders.

In conclusion, In this study, we trained an AI model for the automatic segmentation of the PPs in CT scans to estimate the volume. The segmentations were quantitatively and qualitatively adequate and showed a high correlation to the segmentation of expert readers. Moreover, we showed that higher PPVs are significantly associated with a decreased FVC and VC, but not with DLCO. The AI model is publicly available and can be used to decrease or eliminate the workload for the expert readers, and to study the relation between PPs and lung function more extensively.
